# In Situ Identification of Unknown Crystals in Acute Kidney Injury Using Raman Spectroscopy

**DOI:** 10.3390/nano12142395

**Published:** 2022-07-13

**Authors:** Youjia Yu, Qiaoyan Jiang, Hua Wan, Rong Li, Yang Sun, Zhiwei Zhang, Zhengsheng Mao, Yue Cao, Feng Chen

**Affiliations:** 1 Department of Forensic Medicine, Nanjing Medical University, Nanjing 211166, China; yuyoujia@njmu.edu.cn (Y.Y.); qiaoyanj@126.com (Q.J.); wanhua2006@njmu.edu.cn (H.W.); lirong8321@njmu.edu.cn (R.L.); synjmu@126.com (Y.S.); su311@njmu.edu.cn (Z.Z.); maozhengsheng@njmu.edu.cn (Z.M.); 2 Sir Run Run Hospital, Nanjing Medical University, Nanjing 211166, China; 3 Key Laboratory of Targeted Intervention of Cardiovascular Disease, Collaborative Innovation Center for Cardiovascular Disease Translational Medicine, Nanjing Medical University, Nanjing 211166, China

**Keywords:** Raman spectroscopy, crystal nephropathy, acute kidney injury, burn, pathological diagnosis

## Abstract

Raman spectroscopy is a well-established and powerful tool for in situ biomolecular evaluation. Type 2 crystal nephropathies are characterized by the deposition of crystalline materials in the tubular lumen, resulting in rapid onset of acute kidney injury without specific symptoms. Timely crystal identification is essential for its diagnosis, mechanism exploration and therapy, but remains challenging. This study aims to develop a Raman spectroscopy-based method to assist pathological diagnosis of type 2 crystal nephropathies. Unknown crystals in renal tissue slides from a victim suffered extensive burn injury were detected by Raman spectroscopy, and the inclusion of crystals was determined by comparing Raman data with established database. Multiple crystals were scanned to verify the reproducibility of crystal in situ. Raman data of 20 random crystals were obtained, and the distribution and uniformity of substances in crystals were investigated by Raman imaging. A mouse model was established to mimic the crystal nephropathy to verify the availability of Raman spectroscopy in frozen biopsy. All crystals on the human slides were identified to be calcium oxalate dihydrate, and the distribution and content of calcium oxalate dihydrate on a single crystal were uneven. Raman spectroscopy was further validated to be available in identification of calcium oxalate dihydrate crystals in the biopsy specimens. Here, a Raman spectroscopy-based method for in situ identification of unknown crystals in both paraffin-embedded tissues and biopsy specimens was established, providing an effective and promising method to analyze unknown crystals in tissues and assist the precise pathological diagnosis in both clinical and forensic medicine.

## 1. Introduction

Raman spectroscopy is an well-established and powerful tool for biomolecular evaluation, which provides biochemical information (molecular fingerprints) of samples in vitro and in vivo, without the need for complex sample preparation and staining procedures [1,2,3]. At present, Raman spectroscopy has become an early diagnostic tool, which has been widely used in clinical in vitro and in vivo studies, for the diagnosis of inflammatory diseases and cancers in different organs, as well as unlabeled histopathology and cytology [4,5,6,7]. For example, surface-enhanced Raman spectroscopy methods have been established to monitor endogenous hydrogen sulfide and catalytic process in situ [8,9,10]. Raman technology has several advantages for diagnostic detection of biological samples [11,12,13,14,15,16,17]. Raman spectroscopy relies on inelastic light scattering following the interaction of photons with vibrating molecules that provide sensitive quantitative and chemically specific information about important biological components in the cellular and tissue environment. The energy difference between the incident photons and the inelastic scattered photons is related to the energy required to excite a particular vibration of the molecule. Therefore, biomolecules with distinct chemical and molecular characteristics (such as lipid, DNA and crystal compounds) can be easily identified and quantified without the need for exogenous markers. Changes in these molecular fingerprints can provide disease-specific information. Raman scattering is compatible with near-infrared excitation sources and can be used for real-time measurements, which makes this technique extremely beneficial for biomedical applications, especially in the time-efficient diagnosis. Without the requirement for sample pretreatment processes, sample detection is simple and convenient, meaning that diagnostic costs are low and results can be obtained within minutes. Moreover, the control of Raman spectroscopy conditions ensures nondestructive testing that can obtain accurate results without causing damage to biological samples. Therefore, the application of Raman spectroscopy in identification of unknown components in tissues can be a novel method assisting accurate pathological diagnosis, and provides new insights into the pathogenesis of diseases. 

Acute kidney injury (AKI) is a common complication after severe burns, with morbidity and mortality of up to 30% and 80%, respectively [18]. AKI can easily lead to renal failure and is also an early manifestation of multiple organ dysfunction syndromes (MODS) [19], which contributes to the poor prognosis. Clinically, the reversal of the underlying cause of AKI is usually the first intervention [18]. However, the care of AKI is deemed inadequate in many cases, due to the poor recognition of risk factors [20]. The etiologies of AKI are complicated and vary from factors that directly injure the kidney or cause acute impairment in renal functions [21]. Crystal nephropathy, caused by the deposition of crystalline material in the renal vasculature, the nephron or the draining urinary tract, is among the causes of AKI [22]. Crystal nephropathies can be divided into three subgroups by the classical concept of prerenal, intrarenal and postrenal kidney injury [22]. Among the subgroups, type 2 crystal nephropathies arise from rapid and diffuse intratubular crystallization and cause tubule obstruction, interstitial inflammation and tubular cell injury, resulting in necrosis or apoptosis of tubular cells [23]. Timely diagnosis and exploration of the mechanisms of crystal nephropathy may aid the development of the therapeutic strategy for AKI triggered by crystals. The crystalline materials can be observed by pathological examination, but the identification of the type of crystals mainly relies on morphological observations, which requires experienced pathologists and may lead to misdiagnosis. Therefore, it is essential to develop a fast and accurate method for crystal identification. Raman spectroscopy is a powerful tool with great potential in targeting and monitoring biopsies to obtain information about unknown objects in situ quickly and nondestructively.

In this study, we successfully identified unknown crystals in renal tissue slides in situ as calcium oxalate dihydrate by Raman spectroscopy during forensic pathological examinations in a case of crystal-triggered AKI after severe burn in a young male victim, and, thereby, revealed the potential cause of AKI. Furthermore, we established a mouse model of calcium oxalate dihydrate crystal nephropathy which mimicked the biopsy to verify the availability of Raman spectroscopy in pathological biopsy. Here, we are the first to report the forensic application of Raman spectroscopy in the in situ identification of unknown crystalline substances in tissue slides and further confirm that Raman spectroscopy is also an effective method to analyze unknown substances in biopsy tissue, providing a reliable method and guidance for both clinical diagnosis and postmortem identification of the cause of death.

## 2. Materials and Methods

### 2.1. Case Information

Clinical information was supplied in the supplementary data (Table S1). In brief, an 18-year-old male weighing 57.0 kg, deceased due to multi-organ failure (MOF) 7 days after suffering an extensive flame burn injury. Autopsy and pathological examination were performed one day after death.

### 2.2. Mouse Model

The mouse experiment was designed in accordance with the guidelines of Institute for Laboratory Animal Research of the Nanjing Medical University. All protocols have been approved by the Animal Care and Ethical Committee of Nanjing Medical University (No. IACUC-2107029). Seventeen 8-week male C57/BL6 mice (Oriental Bio Service Inc., Shanghai, China) with 20–25 g body weight (BW) were maintained under a constant environmental condition with temperature 23 ± 2 °C, humidity 55 ± 5%, 12:12 h light/dark cycle in the Animal Research Center of Nanjing Medical University with free access to food and water before experiment. Mice were gavaged with 10 mg/25 g BW oxalic acid (BBI, Shanghai, China) once and sacrificed after 1 h (*n* = 3), 6 h (*n* = 3), 12 h (*n* = 3) or 24 h (*n* = 3), and the other 5 mice were used to evaluate the time of death after gavage.

### 2.3. Tissue Processing and Slide Preparation

The patient’s kidney tissues were fixed in 10% formalin, embedded in paraffin and cut into 5 μm slides. The mice’s kidney tissues were embedded with 30% sodium carboxymethylcellulose (CMC-Na) (Sangon Biotech, Shanghai, China) directly after the mice were sacrificed and were frozen in liquid nitrogen. Then, the frozen blocks were cut into 10 μm slides. Both human and mice slides were stained with routine hematoxylin and eosin (HE). Slides were observed under the optical microscope (Olympus, Tokyo, Japan). For Raman imaging, both paraffin and frozen slides were cut into 20 μm and placed on CaF_2_ discs. The paraffin slides were rehydrated by oxylene for 20 min 3 times, 100% ethanol 15 min twice, 95% ethanol 15 min, 80% ethanol 15 min, 70% ethanol 15 min and ddH_2_O 15 min.

### 2.4. Raman Spectrum and Imaging

Raman spectra and imaging were carried out on Raman Spectrometer—Confocal Raman Microscope (HORIBA, XploRA™ PLUS, Kyoto, Japan). The embedding medium, optimal cutting temperature compound (OCT) and CMC-Na, was placed on a clean silicon chip (Lijing Optoelectronic Technology Co., Ltd., Zhejiang, China), and the detection parameters were as follows: laser wavelength 785 nm, laser power 100 mW, integral time (laser action time) 1 s. Kidney tissue samples were placed on Raman Substrate Materials—CaF_2_ discs, and the CaF_2_ discs were mounted on the XY stage. The area of interest was selected through the bright field image, and the instrument control software was used to automatically collect the Raman spectrum of the tissue. In order to avoid high fluorescence, we chose the excitation wavelength of 785 nm, the laser power of 30 mW, and the spectrum acquisition time of 5 s to achieve sufficient Signal to Noise Ratio without causing any obvious tissue damage. Fast Raman imaging was performed with the 1000 nm step size and 1 s integration time each pixel. The Raman data analysis was performed with Labspec 6 software, and the baseline was subtracted based on polynomial fitting to yield flat background.

## 3. Results

### 3.1. Pathological Findings

**Figure 1:**
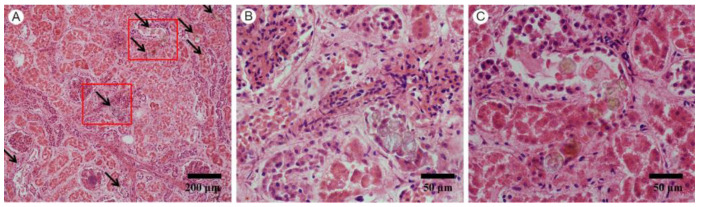
(**A**) Multiple distributions of crystals in renal parenchyma of the patient (HE, magnification 100×); (**B**) a crystal cluster in the renal tubule (HE, magnification 400×); (**C**) crystals were lining up in the renal tubule (HE, magnification 400×).

Microscopic examination of the patient disclosed multiply round brown crystals with double refraction of “Maltese cross” and concentric stripes in the lumen of renal tubules (Figure 1A). These crystals were gathering in clusters or were lining up in renal tubules (Figure 1B,C), especially in distal tubules and collecting ducts. Renal tubular epithelial cells were swelling and necrotic. The postmortem findings suggested crystal nephropathies and supported the clinical diagnosis of MOF.

### 3.2. Raman Detection of Unknown Crystals

**Figure 2:**
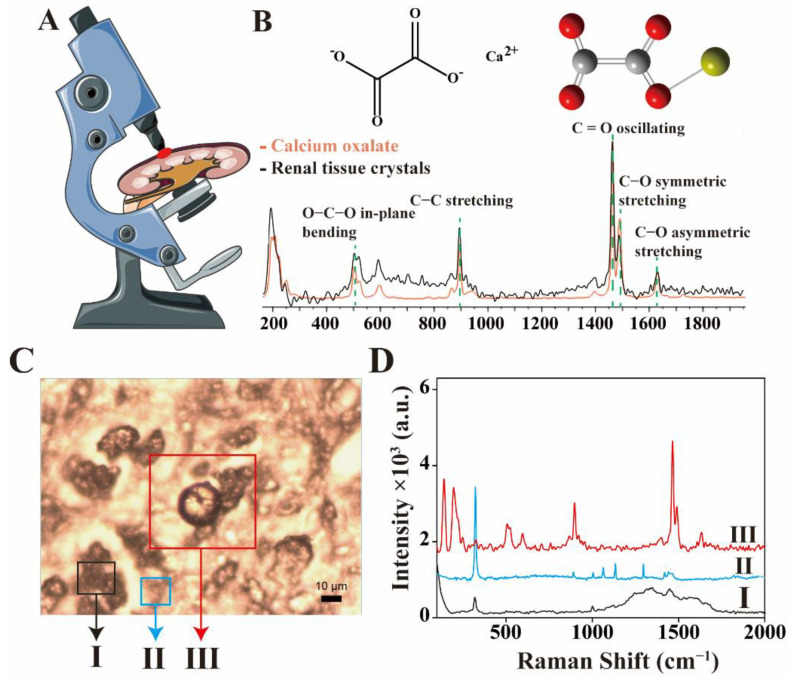
(**A**) Schematic diagram of Raman detection of crystals. (**B**) The measured data were compared with the database of HORIBA and the crystals were calcium oxalate dihydrate. (**C**) Bright field image of a tissue section, the part of kidney tissues (I), the part of paraffin (II) and the part of calcium oxalate dihydrate crystals (III) (magnification 500×). (**D**) Raman spectral data corresponding to (**C**).

The diagram of Raman detection of renal tissue was shown in Figure 2A. Crystals were found under the microscope and Raman spectra were collected. By comparing the Raman spectra with the database of HORIBA, the crystal was identified to be calcium oxalate dihydrate (Figure 2B), with the Raman bands at 506, 897, 1464, 1490 and 1633 cm^−1^, attributing to O–C–O in-plane bending, C–C stretching, C=O oscillating, C–O symmetric stretching and C–O asymmetric stretching, respectively [24,25,26,27].

Raman spectroscopy was performed at different places in the same region of kidney tissue, as shown in Figure 2C. A typical spectrum of calcium oxalate dihydrate was observed from the well-defined crystal (III). Different spectra were found on other structures beside the crystal. The Raman spectrogram of kidney tissue was shown Figure 2D (I). The Raman peak of paraffin was also detected (Figure 2D (II)), indicating that the tissue section was not fully dewaxed. These results suggested that when Raman spectroscopy was carried out on the paraffin embedded tissues, spectra of residual paraffin might be recorded and disturb Raman detection.

### 3.3. Reproducibility and Raman Imaging of Calcium Oxalate Dihydrate Crystals

**Figure 3:**
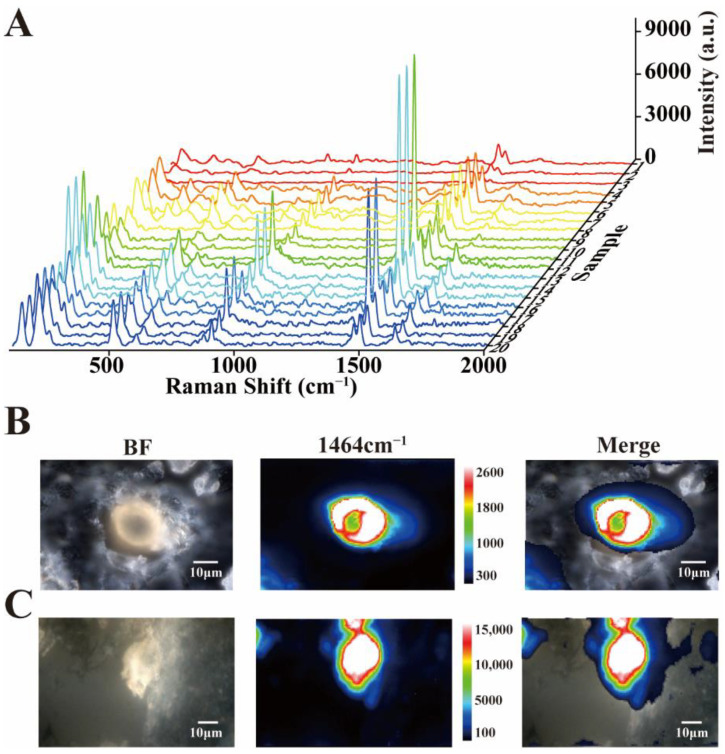
(**A**) Raman spectra of 20 random crystals in tissue sections from the patient. (**B**) Raman mapping of calcium oxalate dihydrate crystals (magnification 1000×). (**C**) Raman mapping of calcium oxalate dihydrate crystals (magnification 600×). BF = bright field.

In order to avoid the complexity of crystal composition and the error of detection results obtained from a single crystal, detection of multiple crystals in the renal tissue was necessary. To test whether all these crystals were calcium oxalate dihydrate, spectra of 20 casually chosen crystals were gathered. According to the Raman spectrum data, although the intensities of 20 crystals were different, they all showed the characteristic Raman spectrum of calcium oxalate dihydrate, while the responding Raman spectra of crystals did not vary visibly (Figure 3A). Crystals were naturally formed in the metabolic process of organisms, so the content and purity of each crystal might not be the same.

The composition and purity of the individual crystals were verified by imaging in different locations. According to the imaging data, the content of calcium oxalate dihydrate in a single crystal was not uniformly distributed. The Raman intensities of some crystals were higher in the periphery than in the center, while others were the opposite (Figure 3B,C), which should be related to the crystal formation mechanism and conditions of calcium oxalate dihydrate crystal. In addition, combining with the bright field images, we could conclude that the bright field images could probably indicate the position and distribution of crystals, but the specific substance identification and contents evaluation needed the assistance of Raman spectroscopy. Raman imaging could clearly determine the contained substances, location distribution and concentration changes of crystals. As shown in Figure 3B,C, the calcium oxalate dihydrate content and distribution of each crystal were different and uneven.

### 3.4. Application of Raman Spectroscopy in Biopsy

As we have proved that the calcium oxalate dihydrate crystals in paraffin embedded tissues could be identified by Raman spectroscopy, we wondered if Raman spectroscopy could be further applied in clinical biopsy samples. Thus, a mouse model of crystal nephropathy was established by oral administration of oxalate to obtain fresh biopsy specimens. To avoid possible disturbance from the embedding medium, we first scanned two commonly used embedding media, OCT and CMC-Na, by Raman spectroscopy. Under the same detection conditions, the Raman peaks of CMC-Na were lower than OCT (Figure S1), indicating that CMC-Na had fewer content substances and chemicals which might cause interference to tissue detection. Therefore, CMC-Na was selected for subsequent tests.

**Figure 4:**
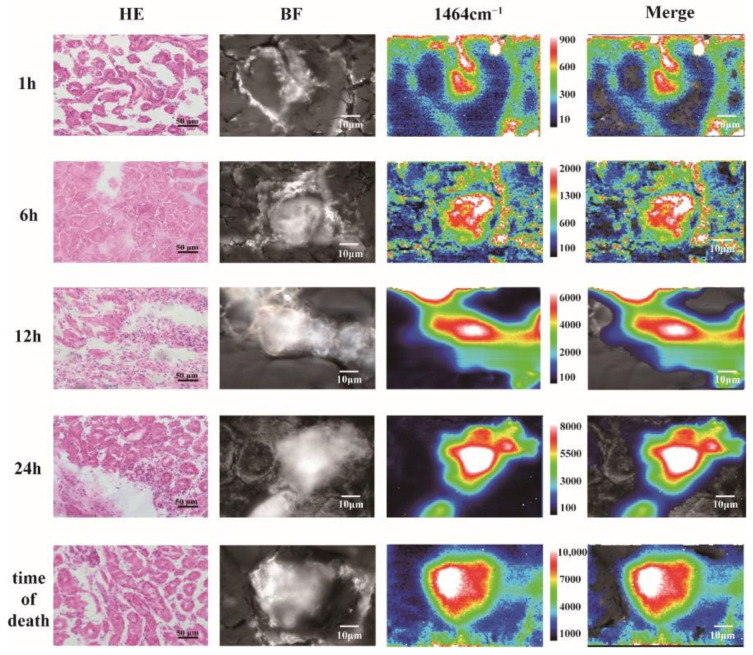
Multiple distributions of crystals with irregular shape in renal tubules of mouse (HE, 400×) and Raman mapping of calcium oxalate dihydrate crystals in the fresh biopsy specimens (magnification 1000×) at different time points (1 h, 6 h, 12 h, 24 h and time of death).

Mice were sacrificed 1 h, 6 h, 12 h or 24 h after gavage with oxalic acid, and all the other 5 mice died after 24~48 h. Kidneys were collected and processed into frozen sections and stained with HE. Under the optical microscope, very few, and tiny, crystals could be found in the mice sacrificed at 1 h. The amount and diameters of crystals increased gradually afterwards. Massive crystals could be found and obstructed in the lumen of renal tubules in mice died after 24 h (Figure 4). In the same way to the detection of calcium oxalate dihydrate crystals in paraffin embedded tissues, we randomly selected 20 crystals on the fresh biopsy specimens (12 h) and collected Raman spectra (Figure S2). The results showed that, regardless of the serial numbers of the crystals, the general spectral characteristics of all the spectra were the same as the characteristic Raman spectrum of calcium oxalate dihydrate. It was normal that some variations presented in the intensities of the Raman spectra, because the content of calcium oxalate dihydrate in different crystals varied. However, the feasibility, accuracy and stability of determination of calcium oxalate dihydrate crystals by Raman spectroscopy would not be affected. Furthermore, the composition and purity of single crystals at different time points (1 h, 6 h, 12 h, 24 h and time of death) were also explored through Raman imaging (Figure 4). The composition of the crystal was calcium oxalate dihydrate, and the distribution of calcium oxalate dihydrate content in the crystal was not uniform. The Raman intensity of the single crystal showed a gradually decreasing pattern from the strongest center to the weak outward. As time went on, the calcium oxalate dihydrate content and diameters of crystals increased gradually afterwards, and the results were similar to HE staining. The location and distribution of calcium oxalate dihydrate crystals obtained through Raman imaging were more intuitive and clearer than the bright field images, without subjective judgment or omission. Comparing to the human samples, oral oxalate administration induced the formation of crystals with purer calcium oxalate dihydrate content in renal tubules, which might be due to a single mechanism of crystal formation. Moreover, besides that paraffin was not used in biopsy samples, no interference of the embedding medium CMC-Na or other interfering substances was found during crystal detection.

## 4. Discussion

In this study, a Raman spectroscopy-based method for in situ identification of calcium oxalate dihydrate crystals in both paraffin-embedded tissues and biopsy specimens was first established and successfully applied to the identification of unknown crystals in the kidneys of a burn victim.

Raman spectroscopy has shown great advantages in the identification of crystals in tissues by maintaining the integrity of the tissue, which can not only obtain more comprehensive and complete useful information, but also allow the tissue to be reused for other detection methods, reducing the amount of required tissue sample, thus limiting the damage to the patient, and costs for sample preparation. Raman spectroscopy provides an instantaneous, convenient and fast approach in crystal identification. Raman data acquired from the tissues are compared with standard graphs in the database, which greatly ensures accuracy and reliability. Moreover, a clear and straightforward boundary of the crystals can be obtained through Raman mapping, making it suitable for determination of locations and distributions of crystals in biopsy samples. Therefore, Raman spectroscopy has important practical significance in clinical application. In our study, Raman spectroscopy is successfully verified to be available for crystal identification in paraffin section, and can be used in both clinicopathology and forensic pathology.

Identification of crystals in biopsy specimens, as well as in paraffin-embedded tissues in postmortem pathological examinations, mainly relies on morphological observations. Calcium oxalate dihydrate crystals show refractivity under polarized light microscopy, which is considered as the characteristic of calcium oxalate dihydrate [22,28]. However, morphological identification requires experience of the pathologist and may be confused with other crystals if the crystals look atypical, especially in the biopsy specimens, which require the pathologist to make diagnosis with quite limited field of microscopic view. In addition, the timely identification of crystals in biopsy specimens can help the clinicians to understand the mechanism of renal dysfunction, and further guide the therapeutic strategy. Thus, to establish a fast and accurate method of crystal identification, Raman spectroscopy was introduced and was validated to be a valuable method in our study. With our method, on one hand, the preparation of the biopsy samples is the same as that used in clinical pathology. Therefore, once the biopsy is required for a patient, we can perform crystal identification by our Raman detection method with the same biopsy specimen, without expending the biopsy wound or gaining more pain to the patient. On the other hand, our method will not occupy much time or increase much work to the pathologists, so that they can still make the pathological diagnosis efficiently. The advantages of Raman spectroscopy in clinicopathology are as follows: (1) the resolution of confocal Ramen spectroscopy is high enough to identify microscopic structures that pathologists need to resolve; (2) comparing with MS equipments, Raman spectroscopy is much cheaper and with a higher resolution and it seems to be impossible to identify a crystal as small as we reported by any MS methods; (3) the Raman spectroscopy method is fast and can be accurate if the database has been established; and (4) the Raman spectroscopy method does not destroy the sample, thus, the sample can be further used in other detection.

Tubular crystallopathies are defined as type 2 crystal nephropathies, and are results of precipitation of minerals, proteins or drugs inside the tubular lumen [29]. There are some common causes of type 2 crystal nephropathies related AKI, for example, drugs, diet and tumor [23]. The occurrence of AKI due to type 2 crystal nephropathy after extensive burn has not been reported yet. Calcium oxalate crystals are mostly reported to be present in diet-induced crystal nephropathy which are metabolites of oxalate or vitamin C-rich foods and drinks [22]. Diseases like short bowel syndrome and genetic disorders, or oxalate and ethylene glycol intoxication may also result in type 2 crystal nephropathy and AKI [22,28,30]. Changes of the microenvironment inside the kidney, such as pH, can be primary causes of calcium oxalate crystal formation [31]. Though impairment of liver and renal functions has been considered a common complication in burn patients, AKI triggered by calcium oxalate dihydrate crystals acted as an exacerbating factor in our case, which was a novel mechanism of AKI after burning injury. By this case, we also would like to highlight the possible value of biopsy in aetiology discovery of AKI patients.

Calcium oxalate is also the most common chemical composition of macroscopic kidney stones that initiates stone formation by crystallization, crystal growth, crystal aggregation, crystal–cell adhesion and crystal invasion through extracellular matrix in renal interstitium [32]. Raman spectroscopy has been successfully applied to analyze mineral components of macroscopic kidney stones that obtained by surgery in some previous studies [24,25,26,27]. In our study of type 2 crystal nephropathy, the crystals in tubules are as small as 10–20 μm in diameter, can only be observed under microscope and cannot be directly isolated from the tissues by any means. Therefore, we utilized confocal Raman microscope for its advantages of high resolution and speediness in the in situ identification of crystals within the tissue slides. Furthermore, our Raman spectroscopy-based method is promising in assisting the diagnosis of type 2 crystal nephropathies caused by other kinds of crystals, as well as type 1 crystal nephropathies, which are caused by micro crystals that obstruct renal vasculature. For the application of our method in clinical and forensic pathology, the next step is to establish the database containing Raman spectra of common crystals in crystal nephropathies and typical samples of different types of crystal nephropathies.

However, attention should be paid to eliminate the interference of paraffin to avoid the inaccuracy of the results. Although the innovative identification of unknown crystals in tissues by Raman spectroscopy can bring advances in clinical diagnosis, a few disadvantages need to be overcome in order to make it a reliable and common diagnostic technique for a wider range of applications. First, the standard Raman spectrum depends on the inelastic light scattering that occurs during the spontaneous process. The spontaneous Raman signal is relatively weak and is often overwhelmed by the elastic light (Rayleigh) scattering signal and the fluorescence of tissues [33,34]. Due to low signal efficiency, spontaneous Raman spectrum often requires a high-power laser, which may cause damage to biological samples, scorching and other irreversible consequences if used under wrong conditions [1]. Second, Raman spectroscopy may not be able to accurately identify and distinguish complex protein structures, and the detection of metabolites may be interfered by some protein components or embedment agents. So it is necessary to introduce specific probes or other diagnostic devices to improve the accuracy and sensitivity of diagnosis [11,13].

## 5. Conclusions

In conclusion, we validate Raman spectroscopy as a fast and accurate method for crystal identification in tissues, which is expected to be applied in clinical and forensic practice in the near future. In view of the successful development of Raman detection method for calcium oxalate dihydrate crystal, we will continue to develop Raman spectroscopy-based methods for the identification of other types of crystals in tissue sections, so as to assist the pathological diagnosis of clinical biopsy and postmortem identification of the cause of death in forensic medicine.

## Data Availability

All data used in this study are available and can be accessed upon reasonable request.

## Supplementary Materials

The following supporting information can be downloaded at: https://www.mdpi.com/article/10.3390/nano12142395/s1, Table S1: Laboratory data; Figure S1: Raman spectra of CMC-Na (a) and OCT (b); Figure S2: Raman spectra of 20 randomly selected crystals in the fresh biopsy specimens (12 h).
