# Supplementary material for: In Situ Identification of Unknown Crystals in Acute Kidney Injury Using Raman Spectroscopy
Source: Nanomaterials (Basel). 2022 Jul 13;12(14):2395. doi: 10.3390/nano12142395 (PMC9323692; doi:10.3390/nano12142395)
Supplement: Supplementary file 1 [file nanomaterials-12-02395-s001.zip › nanomaterials-1764247-supplementary.pdf]

Supplementary Materials

In Situ Identification of Unknown Crystals in Acute Kidney Injury Using Raman Spectroscopy

Youjia Yu [1,†], Qiaoyan Jiang [1,†], Hua Wan [1,2], Rong Li [1], Yang Sun [1], Zhiwei Zhang [1], Zhengsheng Mao [1], Yue Cao [1,*] and Feng Chen [1,3,*]

1 Department of Forensic Medicine, Nanjing Medical University, Nanjing 211166, China; yuyoujia@njmu.edu.cn (Y.Y.); qiaoyanj@126.com (Q.J.); wanhua2006@njmu.edu.cn (H.W.); lirong8321@njmu.edu.cn (R.L.); synjmu@126.com (Y.S.); su311@njmu.edu.cn (Z.Z.); maozhengsheng@njmu.edu.cn (Z.M.)
2 Sir Run Run Hospital, Nanjing Medical University, Nanjing 211166, China
3 Key Laboratory of Targeted Intervention of Cardiovascular Disease, Collaborative Innovation Center for Cardiovascular Disease Translational Medicine, Nanjing Medical University, Nanjing 211166, China
* Correspondence: ycao@njmu.edu.cn (Y.C.); fchen@njmu.edu.cn (F.C.); Tel.: +86-8686-2896 (Y.C.); +86-8686-2896 (F.C.)
† These authors contributed equally to this work.

Table of Contents

1. Clinical information....................S1

2. Table S1....................S1

3. Figure S1-S2....................S2

Clinical information

An 18-year old male weighing 57.0 kg was sent to the emergency department (ED) in a morning 10 hours after suffering an extensive flame burn injury. The patient was mentally confused and complained of thirst upon arrival in the ED. His vitals were as follows: temperature: 37.0℃, pulse rate: 147 beats/min, respiratory rate: 20 breaths/min. Blood pressure was not measured due to severe burn injury on extremities. On physical examination, deep second to third degree burns were observed on the neck, dorsum, buttocks, whole upper extremities and upper part of lower extremities. Around 60% of the total body surface area (TBSA) was affected. His past medical history was unknown. The patient was treated with fluid resuscitation, anti-shock, anti-infection, silver-coated dressings, and other symptomatic treatments. Impairment of liver and renal functions was indicated by laboratory tests during hospitalization (Supplementary Table 1). On the 7th day, anuria, tachypnea and tachycardia occurred. The urine output was reduced to approximately 15 mL/hr. Elevated levels of total bilirubin (63.4 μmol/L), direct bilirubin (42.5 μmol/L), serum creatinine (Cr) (297 μmol/L) and blood urea nitrogen (BUN) (34.3 mmol/L) indicated hepatic and renal injury. But levels of alanine aminotransferase (ALT) and aspertate aminotransferase (AST) decreased to normal ranges comparing with before. The patient passed away due to multi-organ failure (MOF) after 4 hours.

Table S1. Laboratory data.

Parameters	Day 1	Day 4	Day 7
Total bilirubin, μmol/L	41.3	16.7	63.4
Direct bilirubin, μmol/L	0	9.7	42.5
Indirect bilirubin, μmol/L	22.9	7.0	3.6
ALT, U/L	51	27	39
AST, U/L	230	60	43
AST/ALT	4.51	2.25	1.01
Serum creatinine, μmol/L	153	434	297
Blood urea, mmol/L	17.1	30.5	34.3
Potassium, mmol/L	5.38	6.15	3.8

Sodium, mmol/L	135.4	133.7	139.5
Calcium, mmol/L	2.09	1.67	1.72
Phosphorus, mmol/L	2.73	1.83	1.5

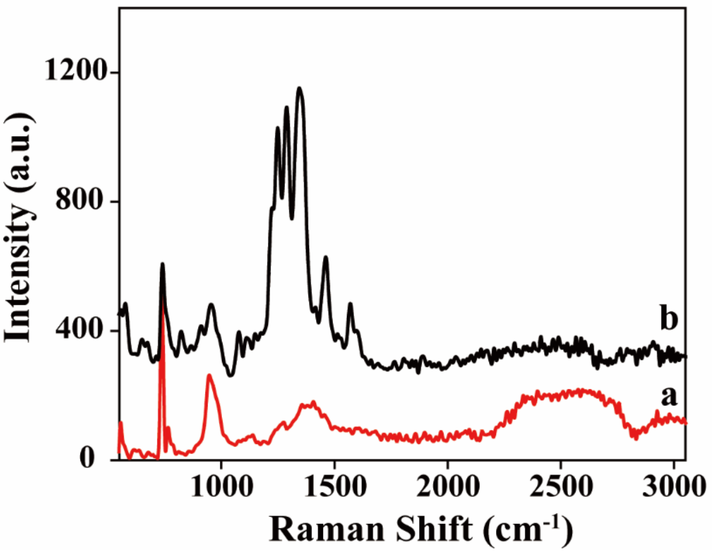

Figure S1. Raman spectra of CMC-Na (a) and OCT (b).

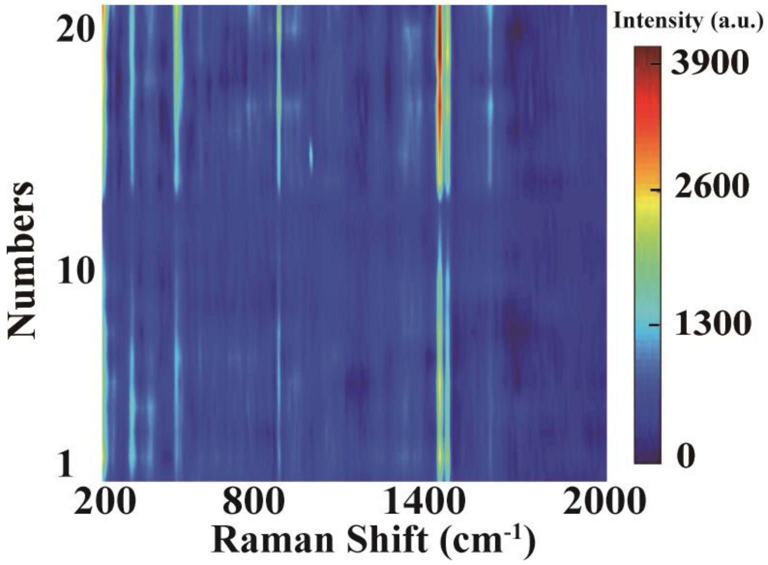

Figure S2. Raman spectra of 20 randomly selected crystals in the fresh biopsy specimens (12 h).
